# Development of a UV-Stabilized Topical Formulation of Nifedipine for the Treatment of Raynaud Phenomenon and Chilblains

**DOI:** 10.3390/pharmaceutics11110594

**Published:** 2019-11-09

**Authors:** Ellen K. Wasan, Jinying Zhao, Joshua Poteet, Munawar A. Mohammed, Jaweria Syeda, Tatiana Orlowski, Kevin Soulsbury, Jacqueline Cawthray, Amanda Bunyamin, Chi Zhang, Brian M. Fahlman, Ed S. Krol

**Affiliations:** 1 College of Pharmacy and Nutrition, University of Saskatchewan, Saskatoon, SK S7N 5E5, Canada; joshua.poteet@usask.ca (J.P.); munawarali89@gmail.com (M.A.M.); jaweriasyeda@hotmail.com (J.S.); tmo380@mail.usask.ca (T.O.); jacqueline.cawthray@fedorukcentre.ca (J.C.); amb902@mail.usask.ca (A.B.); chz855@mail.usask.ca (C.Z.); Brian.Fahlman@gilead.com (B.M.F.); ed.krol@usask.ca (E.S.K.); 2 Faculty of Pharmaceutical Sciences, University of British Columbia, Vancouver, BC V6T 1Z3, Canada; jrzhao0810@gmail.com; 3 British Columbia Institute of Technology, Burnaby, BC V5G 3H2, Canada; Kevin_Soulsbury@bcit.ca

**Keywords:** nifedipine, emulsion, flavonoids, topical formulation, quercetin, photostabilizers

## Abstract

Raynaud’s Phenomenon is a vascular affliction resulting in pain and blanching of the skin caused by excessive and prolonged constriction of arterioles, usually due to cold exposure. Nifedipine is a vasodilatory calcium channel antagonist, which is used orally as the first-line pharmacological treatment to reduce the incidence and severity of attacks when other interventions fail to alleviate the condition and there is danger of tissue injury. Oral administration of nifedipine, however, is associated with systemic adverse effects, and thus topical administration with nifedipine locally to the extremities would be advantageous. However, nifedipine is subject to rapid photodegradation, which is problematic for exposed skin such as the hands. The goal of this project was to analyze the photostability of a novel topical nifedipine cream to UVA light. The effect of incorporating the photoprotectants rutin, quercetin, and/or avobenzone (BMDBM) into the nifedipine cream on the stability of nifedipine to UVA light exposure and the appearance of degradation products of nifedipine was determined. Rutin and quercetin are flavonoids with antioxidant activity. Both have the potential to improve the photostability of nifedipine by a number of mechanisms that either quench the intermolecular electron transfer of the singlet excited dihydropyridine to the nitrobenzene group or by preventing photoexcitation of nifedipine. Rutin at either 0.1% or 0.5% (*w*/*w*) did not improve the stability of nifedipine 2% (*w*/*w*) in the cream after UVA exposure up to 3 h. Incorporation of quercetin at 0.5% (*w*/*w*) did improve nifedipine stability from 40% (no quercetin) to 77% (with quercetin) of original drug concentration after 3 h UVA exposure. A combination of BMDBM and quercetin was the most effective photoprotectant for maintaining nifedipine concentration following up to 8 h UVA exposure.

## 1. Introduction

Raynaud’s Phenomenon (RP) is a vascular condition that causes temporary arteriolar vasospasm in cold-exposed hands and feet of affected persons, resulting in numb, ischemic digits. First, there is a characteristic blanching of the skin as circulation is reduced; secondly, the affected area turns a bluish color during resolution of the vasospasm caused by venous blood returning; and thirdly, redness as arteriolar flow resumes. Not only fingers and toes may be affected, but also the tip of the nose, pinnae of the ears, and the nipples. Rewarming is a painful process. For those seriously affected, RP adversely affects quality of life [1]. Thermoregulatory arteriovenous anastomoses, which are enervated by sympathetic nerves, are responsible for the phenomenon, rather than capillaries, which deliver normal circulation. Connective tissue disease, occupational exposure to vibration, and smoking are risk factors, but RP is typically a primary condition, affecting approximately 3–5% of the population or more, depending on climate [2]. Chilblains is a related cold-induced vascular disorder, resulting in papules causing pain and pruritis [3,4]. RP is not always a benign condition; in severe cases associated with scleroderma, rheumatoid arthritis, and other connective tissue diseases, diabetes, or with certain drug exposures, secondary RP can result in tissue damage due to repeated and prolonged ischemia, requiring medical intervention [5]. When adaptive measures to avoid cold exposure are not effective and pharmacological treatment is required to reduce the impact of severe RP, or chilblains, oral calcium channel blockers are the first-line medications, particularly nifedipine, a dihydropyridine compound [6,7]. Alternatives for severe disease include sildenafil and intravenous prostaglandin analogues [8]. Dihydropyridines bind to L-type Ca_V_1.2 calcium channels [9], and in so doing, effect smooth muscle relaxation including vasodilation of arterioles, the therapeutic target in this case. Other drugs in this pharmacological class include diltiazem, nicardipine, felodipine, amlodipine, and related analogues. Nifedipine has more vascular than cardiac effects [10] and has been demonstrated to have moderate efficacy in the treatment of RP and chilblains [6,11]. Daily oral therapy with nifedipine is not always well-tolerated, however, due to systemic side effects such as dizziness and flushing.

Currently, there is no effective nifedipine topical product marketed for acute RP treatment or prevention of symptoms. Topical application of nifedipine would be advantageous as it would provide a rapid effect on the local tissue while limiting systemic exposure. It is expected that topical nifedipine would be extremely useful for reducing the risk of tissue damage in patients with scleroderma, rheumatoid arthritis, systemic lupus erythematosus, and Sjögen’s syndrome, as a part of combination pharmacological therapy for RP and for those who have outdoor occupations with cold exposure. Furthermore, it is anticipated that topical nifedipine, or topical preparations of other calcium channel blockers or vasodilators, will have utility in the future to augment wound healing [12,13,14] and peripheral vascular insufficiency-related conditions, with a potential role in diabetic ulcer treatment [15,16,17].

**Figure 1:**
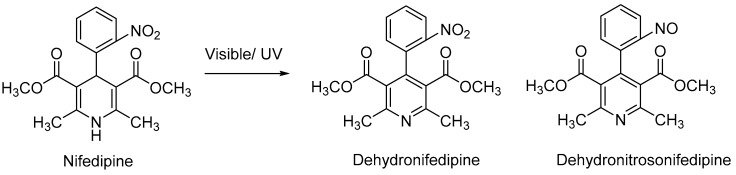
Ultraviolet (UV) radiation-mediated breakdown of nifedipine.

Extemporaneously compounded topical nifedipine has been described, but it has inconsistent efficacy; nifedipine is not stable due to the well-known ultraviolet (UV)-light sensitivity of the drug [18]. Exposure of nifedipine to UVA light (315–400 nm), which accounts for 95% of the UV radiation that reaches the earth’s surface, results in the photodegradation of nifedipine to dehydronifedipine, which can undergo further degradation to form dehydronitrosonifedipine [19,20,21,22,23], both of which are inactive compounds (Figure 1). This degradation process is rapid, it is not sensitive to the presence of oxygen, and it is mainly attributed to UVA irradiation [24,25,26]. One solution to this problem would be to incorporate appropriate photostabilizers; that is, compounds that filter UV energy by absorbing a certain range of high-energy UV wavelengths and releasing the energy at a lower range. We hypothesized that incorporating UV blockers into topical nifedipine formulations would prevent UV-induced decomposition of nifedipine. We describe here a preparation of 2% nifedipine in an oil-in-water emulsion formulation containing photostabilizers that preserves nifedipine from UVA-induced photodegration.

Photostabilization of light-sensitive medications in topical emulsion formulations is not isolated to nifedipine, as a recent analysis of topical products in the United States Pharmacopoeia and the European medicines databases indicated that up to 28% of approved drugs have the recommendation to protect the product from light [27] and the list of new drugs with this recommendation continues to grow [28]. Thus, there is a need for the development of compatible UV blockers for topical formulations. Since topical medications are applied to external body surfaces, they have the potential for significant light exposure. Typically, these preparations are applied as a thin film, which maximizes the surface area of the formulation to UV and visible radiation. In addition to UV or visible light inactivation of topical drug products, other photodegradation products can display toxicities or other unknown effects [29]. Furthermore, light exposure may also influence the physical and technical performance of a topical formulation, such as changes in viscosity, precipitation of components, changes in emulsion droplet size affecting stability, and changes in chemical degradation of materials [27]. Photostabilizers may also serve a role to maintain performance integrity of the topical formulation.

**Figure 2:**
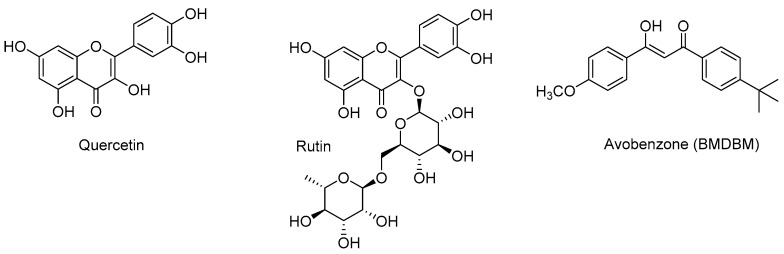
Photostabilizers under investigation in this study.

There are several common photostabilizers that could be appropriate for use in a topical nifedipine formulation including butyl methoxydibenzoylmethane, BMDBM, (an approved sunscreen agent also known as avobenzone) [30,31], and octocrylene, an approved photostabilizer sometimes used in combination with BMDBM in sunscreen products [32]. We have recently been exploring the UV blockers rutin and quercetin, polyphenolic compounds that are found to be upregulated by UV stress in a variety of plant sources [33,34], with known antioxidant and UV-protecting properties [35,36,37,38,39]. Both rutin and quercetin can act as photostabilizers via a number of mechanisms, including preventing photooxidation or inhibiting radical formation, both steps involved in the photodegradation of nifedipine. Additionally, these flavonoids and BMDBM (chemical structures are illustrated in Figure 2) are all characterized by regions of broad absorption that overlap with the absorption of nifedipine, and quercetin has been demonstrated to enhance the photostability of BMDBM in vitro, suggesting that both quercetin and rutin may be suitable photostabilizers [40]. All three can then prevent photodegradation of nifedipine through competitive absorption of photons, thus preventing or minimizing the generation of the first excited state of nifedipine.

Extemporaneously compounded topical nifedipine has been observed to undergo UV-induced decomposition during preparation and storage, contributing to the inactivation and inconsistency of these formulations [18,41]. Nifedipine is not water soluble, which presents certain limitations to the pharmacist such as having to use hydrophobic cream bases or to perform relatively complex compounding procedures. The hydrophobic nature of nifedipine, however, makes the use of an oil-in-water (O/W) emulsion an attractive approach. An added theoretical advantage is the solubility of the photostabilizer compounds in the oil phase of the O/W emulsion, where nifedipine is also solubilized and thereby co-localizing protectant and drug, which may be important for optimal photostabilization. It is important to note that some photostabilizers degrade unless used in combination with other UV blockers. BMDBM has been noted to have sensitivity to UVA irradiation, undergoing photoisomerization to the inactive diketone in non-polar solvents. BMDBM decomposes in aqueous solution, but remains stable in polar solvents [42] and in mineral oil or isopropyl myristate [43]. In order to minimize BMDBM degradation under broad spectrum UV light [UVA plus UVB (280–315 nm)], it is usually used in combination with a UVB blocker or a broad spectrum agent such as octocrylene [32]. We hypothesize that the flavonols quercetin and rutin, through antioxidant and UV absorption properties, will stabilize BMDBM and in turn stabilize nifedipine in our formulation [44,45]. In this report, we have compared quercetin + BMDBM vs. octocrylene + BMDBM on maintaining both BMDBM and nifedipine stability to UVA and UVB light.

## 2. Materials and Methods

### 2.1. Chemicals

Glyceryl monostearate was purchased from Spectrum Industries (Gardena, CA USA). Stearic acid and glycerin were from BASF (Ludwigshafen, Germany). Liquid paraffin, rutin (>94%), quercetin (>95%), and white petrolatum were bought from Sigma-Aldrich (St. Louis, MO USA), and mixed tocopherols from Lotioncrafter.com(Eastsound, WA USA). Sodium lauryl sulphate was from BioRad(Mississauga, ON Canada). Nifedipine (>98%) was from Alpha Aesar (Ward Hill, MA USA). Butyl methoxydibenzoylmethane (BMDBM) was purchased from Tokyo Chemical Industries(Tokyo, Japan). Diethylene glycol monoethyl ether (Transcutol P^®^) was a gift from Gattefossé (Saint-Priest, France). Water was purified by reverse osmosis (MilliQ systemFisher Scientific, Ottowa, ON Canada). Analytical references standards of nifedipine, octocrylene and dehydronitrosonifedipine were from Sigma-Aldrich(St. Louis, MO USA) (99% purity). 

### 2.2. Preparation of Topical Nifedipine

Topical nifedipine was prepared as an oil-in-water emulsion using the beaker method [46]. In general, with this method, the water soluble and oil soluble components are separately dissolved and heated, followed by addition of the water phase to the oil phase with continuous mixing for formation of an emulsion, followed by cooling to solidify the cream. In this case, nifedipine was incorporated into the internal oil phase of the emulsion. All excipients in the formula including glyceryl monostearate, stearic acid, liquid paraffin, petrolatum, diethylene glycol monoethyl ether, glycerin, and sodium lauryl sulfate were used within approved US FDA inactive ingredient levels. The photostabilizer BMDBM was used within the US FDA approved usage level [47]. Flavonoids, rutin, and quercetin were included in the formulation to investigate their potential as UV blockers to facilitate photostabilization of nifedipine in the cream.

Where indicated, when quercetin, BMDBM, or rutin were incorporated into the cream, they also went into the oil phase of the emulsion. Work was conducted under yellow light (577–597 nm), which does not cause photodegradation of the compounds of interest. For the oil phase, glyceryl monostearate (6.7% *w*/*w* of final preparation), stearic acid (9.5% *w*/*w*), liquid paraffin (9.5% *w*/*w*), petrolatum (9.5% *w*/*w*), and Transcutol P (2% *w*/*w*) were weighed into a 250 mL beaker and warmed in a water bath on a hotplate to 85 °C with stirring until homogeneous, followed by addition of the nifedipine (2% *w*/*w*). Where indicated, the following additives were included in the oil phase: quercetin (0.5–2% *w*/*w*), rutin (0.5–2% *w*/*w*), and/or BMDBM (0.5–2%). For the water phase, Milli-Q purified water (q.s.), glycerin (13.4% *w*/*w*) and sodium lauryl sulfate (0.95% *w*/*w*) were warmed in a beaker to 85 °C using a water bath with stirring. The water phase was added slowly to the warmed oil phase with continuous stirring, and within a few minutes, emulsion formation was noted by a visual change to opacity as well as a sudden increase in viscosity. The emulsion in the water bath was removed from heat and stirred continuously at room temperature until reaching 40 °C, followed by homogenization (Virtex23 homogenizer, The Virtex Co., Gardiner, NY USA) for 5 min, then allowed to cool completely at ambient temperature (18–21 °C). Prepared creams were protected from light and stored at 4 °C.

### 2.3. Light Exposure

Photostability tests were conducted in a manner consistent with ICH photostability testing guidelines [48], although conducted in an academic laboratory. Two F20T12/BL/HO UVA lamps (National Biological Corp., Beachwood, OH, USA) filtered to remove UVC with an intensity of 740–750 μW·cm^−2^ at 365 nm as measured with a UVP UVX-36 sensor (Ultraviolet Products Ltd., Upland, CA, USA) were used for irradiation [35]. This level of flux is roughly equivalent to a bright sunny day in mid-summer. Although the lamps used in these studies conform with ICH guidelines, which focus on product/packaging stability (https://www.ich.org/fileadmin/Public_Web_Site/ICH_Products/Guidelines/Quality/Q1B/Step4/Q1B_Guideline.pdf), the focus of our study was on UV-mediated degradation of a topically applied substance with the result that a flux level previously used to mimic topical stability was chosen. The lamp apparatus was placed inside an enclosure with an access door, to prevent ambient light entering and for worker safety. For samples exposed to “ambient light”, these were placed on a laboratory bench where standard fluorescent lighting was used. For UV exposure studies, sample handling was performed under incandescent yellow light to prevent unintentional photodegradation of nifedipine. Nifedipine 20 mg cream samples were spread evenly across the surface of a microscope coverslip to create a thin film. To prevent drying, the samples were covered with Saran Wrap^®^, a plastic film that was determined to be UVA-transparent (data not shown). After the allotted exposure time, the cream was scraped off the slide for extraction with methanol. Samples that were exposed while in solution and not incorporated in a cream were dissolved in methanol as a 20 mL solution in a 100 mL beaker.

### 2.4. Extraction of Nifedipine from the Cream

Solvent extractions were performed under yellow light. The sample was warmed in a water bath to 85 °C to melt lipids, followed by addition of 5 mL of methanol, and vortex mixing. The samples were centrifuged at 10,000 rpm for 5 min, and the supernatant retained for analysis by UV spectrophotometry. The extraction efficiency (EE) was 90%, defined as: EE = (*N*_ex_/*N*_o_) × 100%, where *N*_ex_ is the concentration of nifedipine recovered in the extract and *N*_o_ is the concentration of nifedipine in the original sample, based on the measured amount of nifedipine added.

### 2.5. Spectrophotometric Assay

Nifedipine concentrations were measured in methanol on a UV spectrophotometer (Unico SQ-2800) at 348 nm. The linear range was 5–100 µg/mL (*r*^2^ > 0.999). Values reported represent mean ± SD for triplicate measurements.

### 2.6. Stability Studies

Nifedipine cream was prepared in replicates of 50 g batches and stored at ambient (21 °C) or refrigerated (4 °C) temperatures, protected from light. At the indicated timepoints, triplicate samples of 1 g were removed and extracted as described above, followed by HPLC analysis.

### 2.7. HPLC Assay

Nifedipine was quantified by reverse-phase HPLC at ambient temperature (23 °C) by an isocratic method on a Waters 2690 instrument equipped with a photodiode array detector (Waters 996, Waters Canada, Mississauga, ON Canada). The column was a C18 5 µm 4.6 × 150 mm (Phenomenex, Torrance, CA USA) and the mobile phase consisted of acetonitrile: Sodium acetate (1 mM, pH 5.3 adjusted with HCl) (70:30 *v*/*v*), generating a retention time of 2.9 min for nifedipine (*λ* = 348 nm; linear range 10–100 µg/mL, *r*^2^ > 0.99).

### 2.8. Emulsion Phase Stability Analysis

A high-end Dispersion Analyzer [LUMiSizer^®^ (LUM Corp., Boulder, CO USA)], which is a multi-sample, temperature-controlled analytical photocentrifuge with dedicated software, was used to predict long-term stability and optimization of nifedipine emulsions by means of creaming rate. This allows for an approximation of the relative stability to phase separation of emulsions that differ in the type or percentage of photoprotectant and for confirming batch-to-batch consistency in stability against phase separation. For each sample type, triplicate samples of 2 mL were loaded into photocentrifuge acrylic cuvettes. The samples were centrifuged at 42 °C × 12 h. Phase separation was detected as an increase in light transmission at the top of the sample, which is interpreted as an “instability index” reflecting the rate of change of light transmission.

### 2.9. High-Performance Liquid Chromatography–Photodiode Array (HPLC-PDA) For Ketoprofen Sparing

HPLC-PDA analysis was carried out at room temperature using either a Waters 2695 separation module equipped with a Waters 2996 photodiode array detector (Waters, Milford, MA, USA), or an Agilent Series 1200 quaternary pump (G1311A) with online degasser (G1322A), autosampler (G1329A), and photodiode array detector (G1315D) (Agilent Technologies, Mississauga, ON, Canada). Aliquots were injected onto a 250 × 4.6 mm Allsphere ODS-2 column, 5 μm particle size (Alltech, Calgary AB, Canada). Data were processed using Empower software (Waters, Milford, MA, USA) or Chemstation software (Agilent Technologies, Mississauga, ON, Canada). Elution was carried out in gradient mode using two components: A = 1% formic acid in water, B = 1% formic acid in methanol (flow rate 1 mL/min). The gradient for the UVA experiments was as follows: 5 to 15 min linear gradient from 90% A to 10% A; 15 to 19 min, isocratic 10% A; 19 to 22 min, linear gradient from 10% A to 90% A; 22 to 25 min, isocratic 90% A. 

### 2.10. Time Course for UV Irradiation of Rutin and Ketoprofen

For photostability analysis, a 20 mL solution of rutin (50 µM) or ketoprofen (250 µM) in methanol was placed in 50 mL quartz cells fitted with a septa. The quartz cells were exposed to UVA (740 μW·cm^−2^ at 365 nm). For the methanol experiments, aliquots of 100 μL were taken in duplicate at each time point and injected directly on the HPLC. The time-course samples were compared to the 0 h time point to determine the amount of compound remaining by measuring peak area at the *λ*_max_ for each compound. Experiments were performed on at least 3 separate occasions.

### 2.11. Ketoprofen Sparing

A 20 mL solution of flavonol (50 µM) or ketoprofen (250 µM) in methanol was placed in 50 mL quartz cells fitted with a septa. The quartz cells were exposed to UVA (740 μW·cm^−2^ at 365 nm). Aliquots of 100 μL were taken in duplicate at each time point and injected directly on the HPLC. The time-course samples were compared to the 0 h time point to determine the amount of ketoprofen and flavonol remaining by measuring peak area at the *λ*_max_ for ketoprofen and each flavonol. Experiments for each flavonol were performed on at least 3 separate occasions.

### 2.12. Mass Spectrometry Analysis of Nifedipine and its Photo-degradants

The high-performance liquid chromatography (HPLC) MS/MS system consists of an Agilent series 1200 quaternary pump with an online degasser, auto sampler set to 4 °C, and DAD detector scanning between 190 to 400 nm (Agilent Technologies, Mississauga, ON, Canada ) coupled to an AB Sciex API 4000 QTRAP mass spectrometer. Photodegradants of nifedipine were identified following direct infusion of 2.5 ng by observation of the appearance of the protonated and unprotonated product ions for dehydronifedipine [M]^1+^ to [M^−^ C_17_H_16_N_2_O_6_]^+^ (*m/z* 345 and 344) and for dehydronitrosonifedipine [M]^1+^ to [M^−^ C_17_H_16_N_2_O_5_]^+^ (*m/z* 329 and 328); peak areas were integrated by Analyst Software v1.6 (SCIEX, Redwood City, CA, USA) [43].

### 2.13. Statistical Analysis

Descriptive statistics were generated in Microsoft Excel (Office 2016). Comparison of means was analyzed by ANOVA with Tukey’s post-hoc test (Astatsa, 2016).

## 3. Results and Discussion

The goal of this study was to assess the ability of two polyphenolic flavonols, quercetin and its 3′-rutinoside analog rutin, for their ability to attenuate UVA radiation-mediated decomposition of BMDBM and nifedipine in a topical formulation for the treatment of Raynaud Phenomenon. In order to accomplish these goals, we developed oil-in-water (O/W) emulsions containing mixtures of nifedipine, BMDBM, and either quercetin or rutin. We then exposed the nifedipine-containing emulsions to UVA radiation and employed UV spectroscopy, HPLC, and mass spectrometry to assess the ability of quercetin and rutin to act as photostabilizers.

### 3.1. UVA and Visible Decomposition of Nifedipine

#### Nifedipine in Methanol

**Figure 3:**
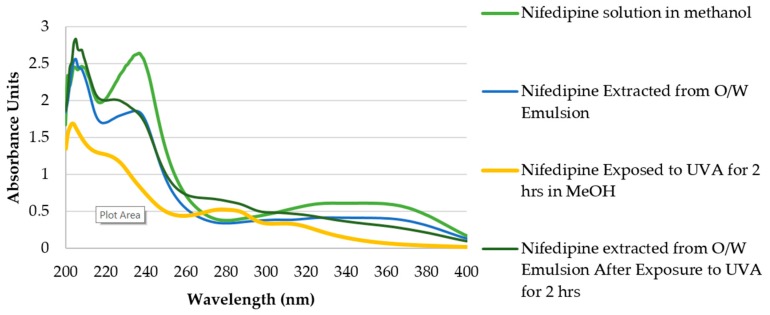
Ultraviolet (UV) absorption spectra of nifedipine as: Nifedipine solution 40 µg/mL in methanol; methanol extract of nifedipine cream (2% (*w*/*w*) as O/W emulsion); nifedipine 40 µg/mL solution in methanol after 2 h exposure to UVA light at a flux of 750 µW/cm^2^; methanol extract of nifedipine cream (2% (*w*/*w*) as O/W emulsion) that was exposed for 2 h to UVA light at a flux of 750 µW/cm^2^ prior to extraction.

The absorption spectrum of nifedipine (Figure 3) is characterized by strong absorbance at 240 nm and a broad absorption peak near 350 nm. UV spectroscopy clearly shows the change in the absorption spectrum of nifedipine in methanol after exposure to UVA radiation for 2 h with a decrease in absorbance at both 240 and 350 nm and new absorption maxima appearing at 280 and 310 nm. These are consistent with the results of Fasani et al. [49] who observed similar spectral changes for nifedipine in ethanol following UV irradiation and others who have noted rapid degradation of nifedipine in methanol solution exposed to laboratory light [50].

### 3.2. Characterization of Emulsion

**Figure 4:**
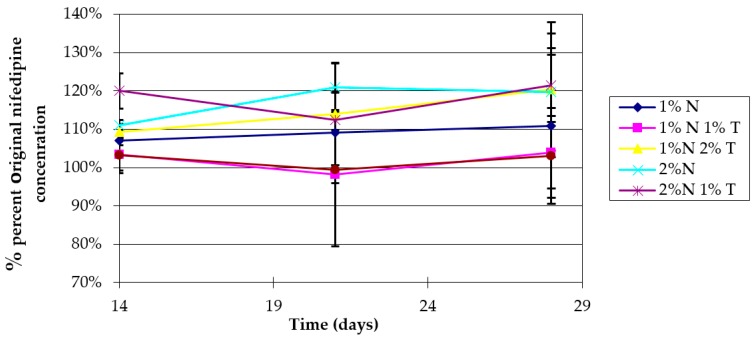
Stability of nifedipine (N) 1% or 2% (*w*/*w*) cream prepared with or without Transcutol HP (T) (1% or 2% (*w*/*w*)). The cream was stored protected from light at ambient temperature (23 °C). At 14, 21, and 28 days, nifedipine concentration was measured by UV spectrophotometry and reported as percent of original concentration. Data represent mean ± SD (*n* = 3).

We chose to prepare a 2% (*w*/*w*) oil in water (O/W) emulsion as our topical delivery vehicle because nifedipine is readily incorporated into the internal oil phase, while a non-greasy feel is still achieved. This is advantageous for patient acceptability, as an oily topical preparation is not desirable for use on the hands and feet. In addition to the photostabilizers, the prototype nifedipine formulations contain the approved topical drug penetration enhancer diethylene glycol monoethyl ether (Transcutol HP^®^). Transcutol HP^®^ is used in these formulations because of its established safety record [51,52], regulatory approval for human use, and ease of incorporation into the emulsions. Nifedipine stability in the cream prepared as 1% or 2% (*w*/*w*) nifedipine with or without Transcutol HP penetration enhancer (1% or 2% *w*/*w*) was determined under light-protected conditions at 23 °C and found to be maintained at >95% of original concentration for at least one month (Figure 4).

**Figure 5:**
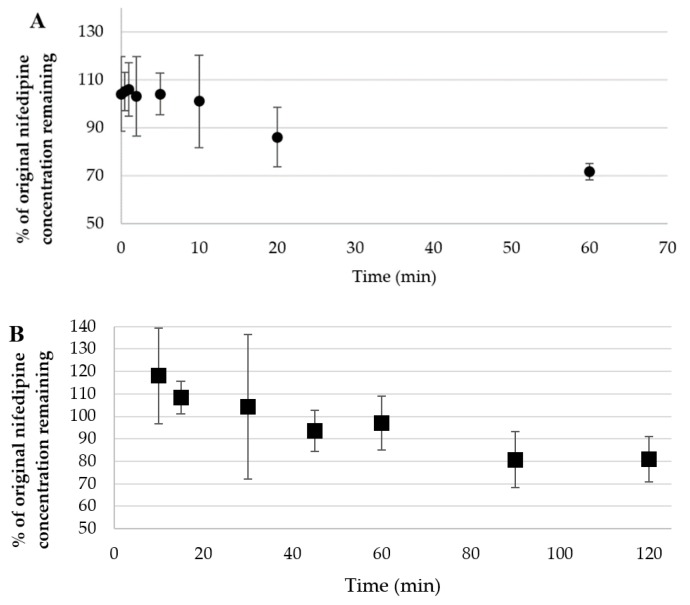
Nifedipine (2% *w*/*w*) cream exposed as a thin film to (**A**) ambient light over 1 h; (**B**) UVA light over 2 h. Data represent concentration vs. time (mean ± SD (*n* = 3)).

A methanol extract of nifedipine cream (2% (*w*/*w*) as O/W emulsion) shows a similar spectrum as observed when dissolved in methanol. Upon exposure of a thin film of the O/W emulsion to indoor fluorescent light (ambient laboratory light), nifedipine concentration began to decline at 20 min, and was reduced to 75% of its original concentration by 1 h (Figure 5A). Formation of degradation products was determined by the appearance of a new absorbance maximum at 280 nm, consistent with formation of the aromatic groups in the degradation products and HPLC and mass spectrometric analysis. We compared APCI/ESI (+) mass spectrometry results (discussed below) with literature values to confirm the identity of the decomposition products (dihydronifedipine: *m/z* 345.10, [M+H]^+^ and dehydronitrosonifedipine: *m/z* 329.11, [M+H]^+^) [53]. Dehydronitrosonifedipine (DHN), the major degradation product, began to appear by 1 h of exposure to visible light. UVA exposure (750 µW/cm^2^) of a thin film of the O/W emulsion over 2 h also resulted in loss of nifedipine (Figure 5B), although minimal degradation occurred in the first 45 min of exposure. After 2 h, 72 ± 7.86% of the nifedipine remained, suggesting that the O/W emulsion imparts some, albeit incomplete, photoprotection of nifedipine to UVA radiation.

### 3.3. Characterization of Topical Nifedipine Cream

#### 3.3.1. Influence of Butyl methoxydibenzoylmethane (BMDBM), Quercetin, and Rutin on Nifedipine UV Stability

**Figure 6:**
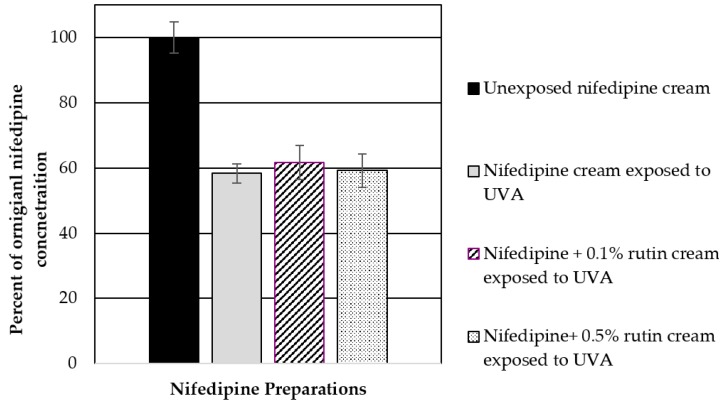
Rutin (0.1% and 0.5% (*w*/*w*)) was incorporated into the nifedipine cream and evaluated for its ability to decrease nifedipine degradation in the cream due to UVA exposure over 3 h. Data represent mean ± SD (*n* = 3).

In an effort to further improve nifedipine stability to light, we assessed the ability of three photostabilizing agents, rutin, BMDBM, and quercetin, either alone or in combination in our O/W emulsions. The photostabilizers were incorporated at varying concentrations up to 3% *w*/*w* in the emulsion and then treated with UVA radiation. Unfortunately, none of the photostabilizers when used on their own were effective in preventing UVA radiation-mediated decomposition of nifedipine when exposed as a thin film. Incorporation of rutin at concentrations of 0.5–1% (*w*/*w*) showed insufficient protection in reducing UVA-induced degradation of nifedipine over 3 h (Figure 6). No further studies were done using rutin as an additive.

**Figure 7:**
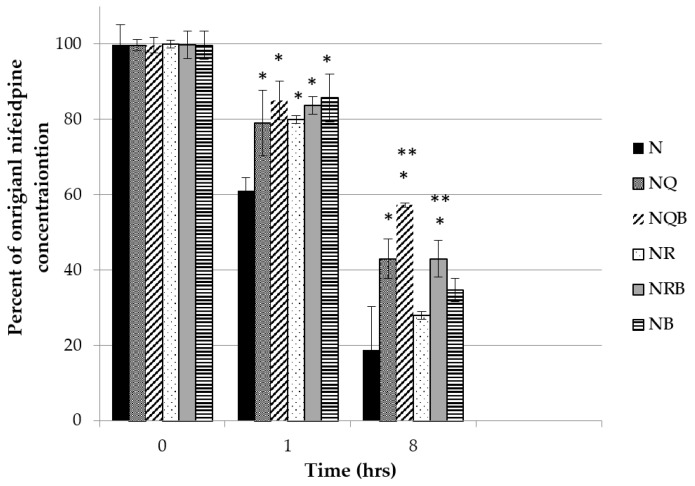
Quercetin or rutin (0.5% (*w*/*w*)) ± avobenzone (BMDBM) (3% (*w*/*w*)) were incorporated into the nifedipine 2% (*w*/*w*) cream as photoprotectant(s). The cream was exposed as a thin film to UVA light. Data represent percentage of original nifedipine concentration vs. exposure time (mean ± SD, *n* = 3). N: Nifedipine only; NQ: Nifedipine with quercetin; NQB: Nifedipine with quercetin and BMDBM; NR: Nifedipine with rutin; NRB: Nifedipine with rutin and BMBDM; NB: Nifedipine with BMDBM. *Significantly different from N (*p* < 0.01); **significantly different from its counterpart without BMDBM (*p* < 0.05).

Similarly, quercetin or rutin at 0.5% (*w*/*w*) or BMDBM alone were not effective as single agents in protecting nifedipine from degradation. A combination of quercetin at 0.5% (*w*/*w*) and BMDBM 3% (*w*/*w*), however, provided the best protection from UVA radiation-mediated decomposition in terms of the original nifedipine concentration maintained after 8 h of UVA exposure (Figure 7).

**Figure 8:**
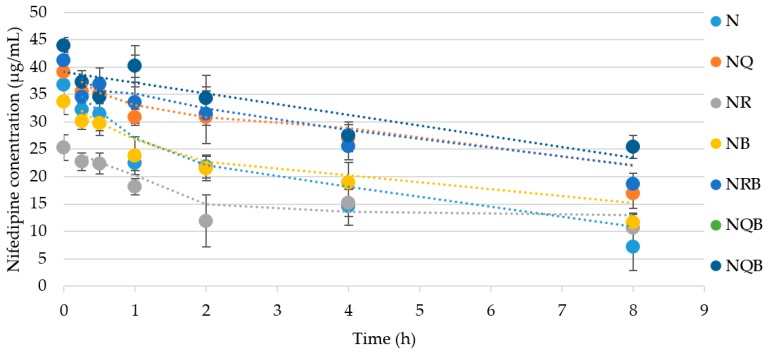
Quercetin or rutin (0.5% (*w*/*w*)) ± BMDBM (3% (*w*/*w*)) were incorporated into the nifedipine 2% (*w*/*w*) cream as photoprotectant(s). The cream was exposed as a thin film to UVA light. Data represent percentage of original nifedipine concentration vs. exposure time (mean ± SD, *n* = 3). N: Nifedipine only; NQ: Nifedipine with quercetin; NQB: Nifedipine with quercetin and BMDBM; NR: Nifedipine with rutin; NRB: Nifedipine with rutin and BMBDM; NB: Nifedipine with BMDBM.

Figure 8 illustrates the degradation profile for each combination, demonstrating a two-phase process where the rate of degradation for the first two hours is greater than the rate for the period of 2–8 h. Table 1 lists the first (0–2 h) and second (2–8 h) phase rates, which suggest that the photoprotectants have the most significant effect on reducing the rate of degradation during the first two hours where the rate *k* is defined as:

(%*N*_t1_ − %*N*_t2_)/(*t*_2_ − *t*_1_)

where %N is percentage of original unexposed nifedipine concentration in the indicated O/W cream formulation and *t*_1_ and *t*_2_ represent exposure timepoints. Thus, in Table 1, *k*_1_ represents the rate of degradation in the first 2 h, and *k*_2_ the rate for 2–8 h of continuing exposure.

Again, the combination of quercetin with BMDBM is shown to be the most effective at reducing the rate of photodegradation of nifedipine in the emulsion.

**Figure 9:**
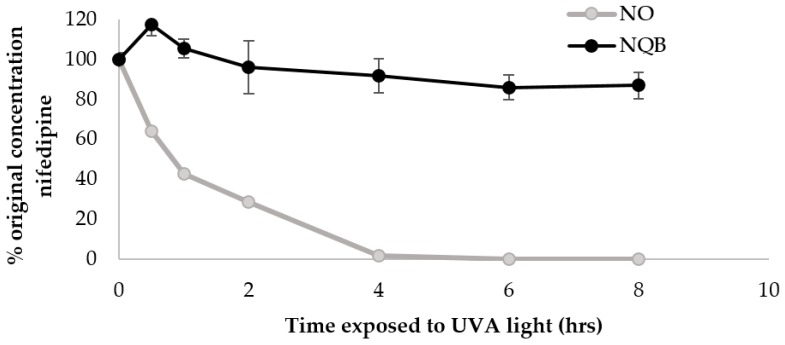
Nifedipine cream was prepared with quercetin 0.5% *(w*/*w*) + BMDBM 3% (*w*/*w*) (NQB) or with octylcrylene 2% (*w*/*w*) (NO) as photoprotectants. The cream was exposed as a thin film to UVA light. Data represent percentage of original nifedipine concentration vs. exposure time (mean ± SD, *n* = 3).

Octocrylene (2% *w*/*w*) was also tested as a potential photoprotectant for nifedipine topical emulsion, prepared in the same way as described above, but it did not prevent nifedipine degradation under UVA exposure (Figure 9). Octocrylene (2% *w*/*w*) with BMDBM (3% *w*/*w*) in combination in the nifedipine cream was not physically stable in the O/W emulsion and was not further optimized.

**Figure 10:**
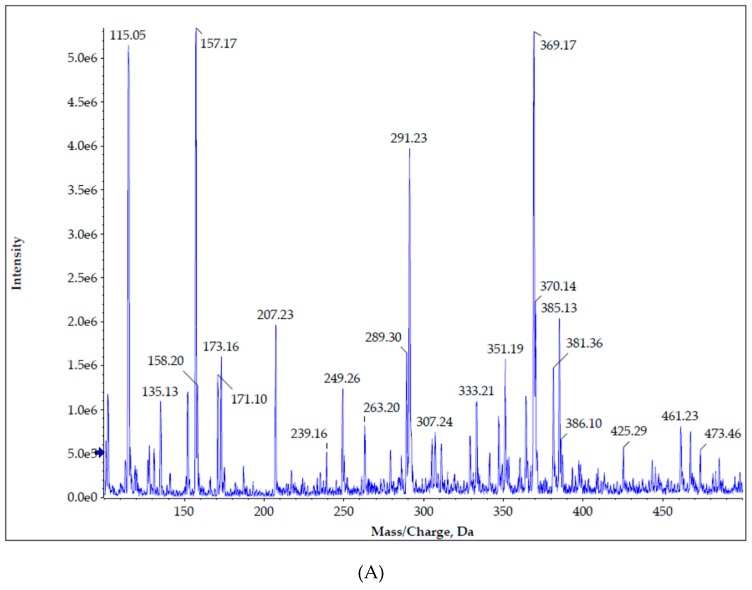

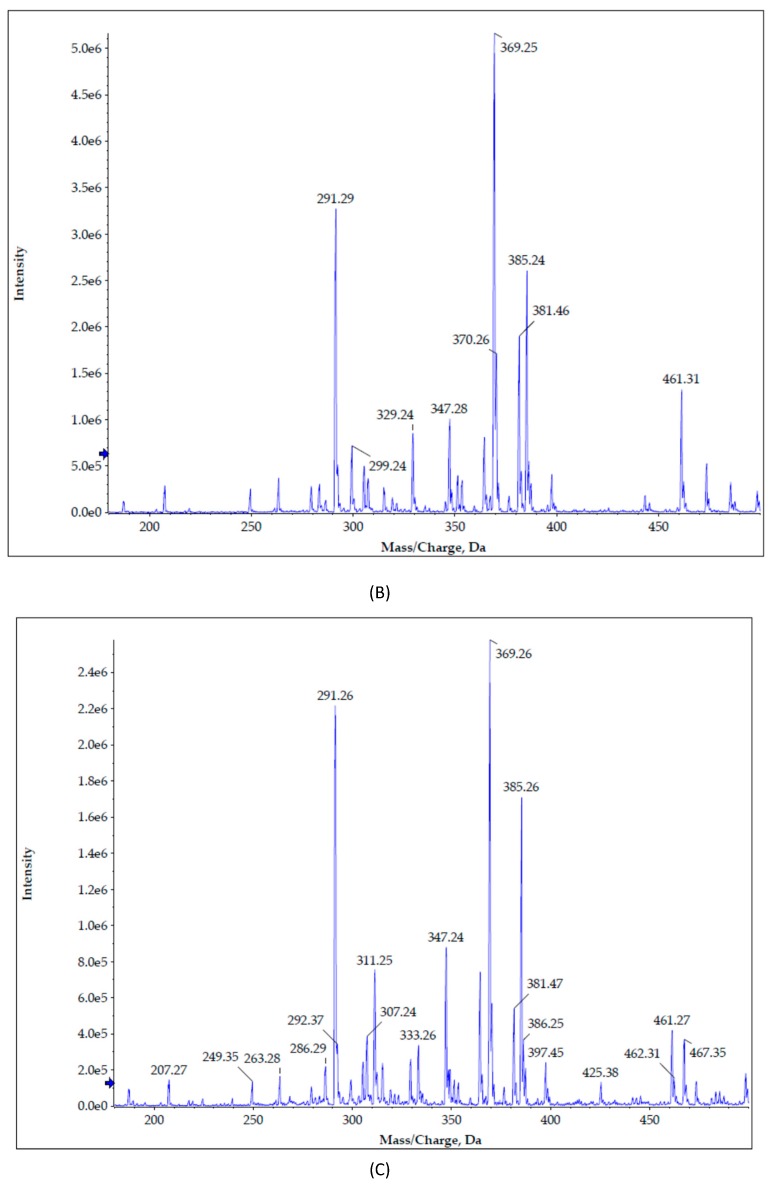
Mass spectrometry shows differential appearance of photodegradation product comparing (**A**) nifedipine 2% cream containing 0.5% quercetin with no UV exposure; (**B**) nifedipine 2% cream containing 0.5% quercetin that was exposed to UVA × 2 h; (**C**) nifedipine 2% cream containing 0.5% quercetin and 3% BMDBM that was exposed to UVA × 2 h.

We used mass spectrometry to determine if the same nifedipine degradation products are formed following UV exposure when one or more of the photostabilizers (rutin, quercetin, BMDBM) is present. Dehydronifedipine and dehydronitrosonifedipine were found in UVA-exposed creams as expected [22], with no alternative degradation pathways identified in the presence of these photostabilizers based on appearance of *m/z* consistent with their expected profiles. A peak for the parent nifedipine (*m/z* of 347 [M]+ and *m/z* of 369.3 [M + Na^+^]^+^) can be seen in all panels of Figure 10. The mass spectrum of nifedipine is shown in supplementary Figure S2. The appearance of *m/z* = 329 is consistent with dehydronitrosonifedipine formation and was previously identified as the principal degradation product by HPLC. The mass spectrum of pharmaceutical reference standard dehydronitrosonifedipine is shown in supplementary Figure S3. The nifedipine was not protected from photodegradation by rutin, and the product *m/z* = 329 again made an appearance. Further investigation of the various photoprotectants indicated no unexpected fragment ion formation (data not shown) to indicate any alternate degradation pathways in the presence of rutin, quercetin, or BMDBM. The three panels of Figure 10 shows differential appearance of presumed photodegradation product *m/z* = 329 comparing (A) nifedipine 2% cream containing 0.5% quercetin; (B) nifedipine 2% cream containing 0.5% quercetin that was exposed to UVA × 2 h at 450 µW/cm^2^; (C) nifedipine 2% cream containing 0.5% quercetin and BMDBM 3% that was exposed to UVA × 2 h at 450 µW/cm^2^. The presence of product ion *m/z* = 329 is consistent with the HPLC results, which analyzed nifedipine and dehydronitrosonifedipine concentration vs. exposure time, as discussed above, although it is acknowledged that secondary MS/MS analysis will be needed to confirm the identity of *m/z* = 329.

**Figure 11:**
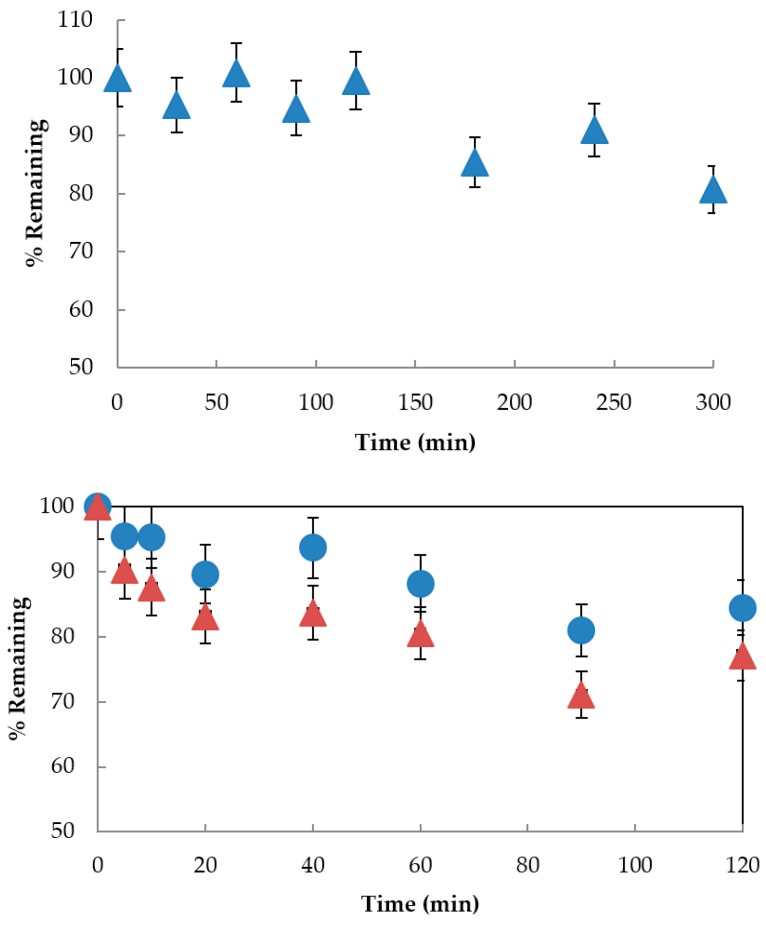
Time-course decomposition for UVA (740 μW·cm^−2^ at 365 nm) exposed compounds in MeOH: (top) (Δ) ketoprofen (250 µM); (bottom) (Δ) ketoprofen (250 µM) in the presence of (●) rutin (50 µM). Data are the mean ± standard deviation of three separate experiments as determined by HPLC-PDA at the *λ*_max_ for ketoprofen and rutin and are reported as % remaining.

The reason behind the inability of two closely related flavonoids to act as photoprotectants was unclear. The decomposition of quercetin in methanol to three major products when exposed to UVA has been described previously [54], however when we examined rutin for UVA stability in methanol, we observed minimal decomposition (Supplementary S1). Furthermore, quercetin has been shown to protect the non-steroidal anti-inflammatory ketoprofen from UV decomposition in vitro [55]. We examined whether rutin could also prevent ketoprofen degradation in an in vitro system. We first confirmed that quercetin could spare UVA-induced ketoprofen degradation by irradiating a methanol solution of ketoprofen with UVA in the presence of quercetin. Consistent with previous reports [55], we observed a loss of quercetin over time and formation of quercetin degradation products, whereas ketoprofen loss was minimal until all of the quercetin had been depleted. Conversely, UVA irradiation of ketoprofen in methanol in the presence of rutin resulted in a more rapid loss of ketoprofen comparable to control exposures and concurrent decomposition of rutin (Figure 11). Since the only structural difference between quercetin and rutin is glycosylation at the 3′−OH of rutin, this suggests to us that 3′−OH substitution confers some UV stability to flavonols. Both quercetin and rutin possess a catechol moiety in the B-ring that undergoes oxidation to an *ortho*-quinone, which in the case of quercetin, appears to lead to photodegradation [54]. This may imply that the decomposition of quercetin is associated with its photoprotective properties; the lack of a photostabilizing effect of rutin on BMDBM is in agreement with these observations.

#### 3.3.2. Effect of Photostabilizers on Emulsion Properties

**Figure 12:**
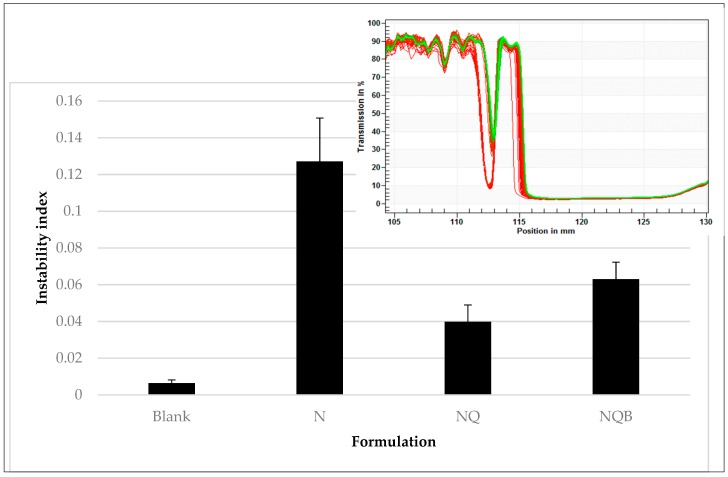
Sedimentation analysis of nifedipine emulsions (N) containing quercetin (NQ) or quercetin plus BMDBM (NQB) show differences in their tendency to exhibit phase separation (at 42 °C over 12 h), as shown by an increase in light transmission at the top of the sample as the oil phase separates to the top. A shift from red to green indicates a change in light transmission between the first and last readings (example shown in inset). This rate of change in light transmission of the sample vs. time is translated into an “instability index”.

Emulsion composition and viscosity are key features to minimize the potential for phase separation. With our observation that a combination of quercetin and BMDBM can protect nifedipine from UVA photodegradation, we next sought to assess whether the photostabilizers might reduce physical stability of the emulsion by affecting viscosity or emulsion droplet formation. To accomplish this, the creaming rate and extent were assessed in a temperature-controlled photocentrifuge whereby an increased transmission to light indicates phase separation, which is then calculated as an instability index. If an excipient caused a significant change in viscosity, for example, phase separation would occur more quickly and adversely affect emulsion stability on storage. This information can drive the decision to choose between two excipients that are otherwise performing similarly. In our system, the incorporation of quercetin or BMDBM reduced the instability of the nifedipine emulsion (Figure 12 and inset, showing differences between emulsion stability depending on presence of specific photostabilizers), possibly by altering the emulsion viscosity. The improved emulsion stability, together with nifedipine photostabilization, suggest that a topical formulation of nifedipine containing quercetin and BMDBM may be an effective approach for local delivery of nifedipine for RP.

## 4. Conclusions

Topical delivery of nifedipine as a treatment for Raynaud’s Phenomenon requires a photoprotectant to prevent nifedipine degradation upon exposure to UV radiation. A combination of the flavonoid quercetin and BMDBM in an O/W emulsion was able to protect nifedipine in vitro from UVA radiation-induced decomposition, whereas rutin in combination with BMDBM did not. Although quercetin and rutin share similar scaffolds, only quercetin was able to act as a photostabilizer of BMDBM and nifedipine, the difference in photostabilizing properties would appear to be a function of the unsubstituted 3′−OH in quercetin. Smith et al. [56] suggested that functionalization of the 3′−OH, or absence of a 3′−OH as in luteolin, confers photostability and that the instability of flavonols with an unsubstituted 3′−OH is proposed to be the result of an excited state electron transfer step, although how this would lead to photoprotection is unclear. Rather, it may be that quercetin more readily directs absorption of UV radiation to degradation, thus sparing BMDBM, whereas rutin does not have a comparable path and may form an excited state which then activates BMDBM. These observations provide insight into the development of quercetin or other flavonoids as photoprotectants for nifedipine or other pharmaceutical agents, which possess UV sensitivity. Follow-up studies will include investigation of the effect of this topical nifedipine formulation on vasodilation in vivo.

## Figures and Tables

**Table 1:** Rate of nifedipine degradation vs. time in O/W formulations containing photoprotectants as described in Figure 8. *Significantly different from formulation N (*p* < 0.5) within that phase. #significantly different from formulation NB (*p* < 0.5) within that phase.

FORMU-LATIONS	Phase 1 0–2 h *k_1_* (%/h)	Phase 2 2–8 h *k_2_* (%/h)
N	14.3 ± 0.8	2.18 ± 0.7
NQ	8.21 ± 2.6* #	2.32 ± 0.49
NR	8.59 ± 2.87	3.5 ± 0.52
NB	7.86 ± 5.1	4.6 ± 0.46 *
NRB	5.26 ± 1.6 *	2.1 ± 0.26
NQB	3.7 ± 2.14 * #	1.4 ± 0.72*

## Supplementary Materials

The following are available online at https://www.mdpi.com/1999-4923/11/11/594/s1, Figure S1: UV absorption spectra of quercetin and rutin before and after UVA exposure. Figure S2. Mass spectrum of nifedipine extracted from the cream (Q1 scan) indicating *m*/*z* = 369. Figure S3. Mass spectrum of dehydronitrosonifedipine (+MRM) indicating *m*/*z* = 329.
