# Supplementary material for: Development of a UV-Stabilized Topical Formulation of Nifedipine for the Treatment of Raynaud Phenomenon and Chilblains
Source: Pharmaceutics. 2019 Nov 9;11(11):594. doi: 10.3390/pharmaceutics11110594 (PMC6920966; doi:10.3390/pharmaceutics11110594)
Supplement: Supplementary file 1 [file pharmaceutics-11-00594-s001.pdf]

pharmaceutics

MDPI

Development of a UV-Stabilized Topical Formulation of Nifedipine for the Treatment of Raynaud Phenomenon and Chilblains: Supplementary Information

Ellen K. Wasan, Jinying Zhao, Joshua Poteet, Munawar Mohammed, Jaweria Syeda, Kevin Soulsbury, Jacqueline Cawthray, Amanda Bunyamin, Chi Zhang, Brian M. Fahlman and Ed S. Krol

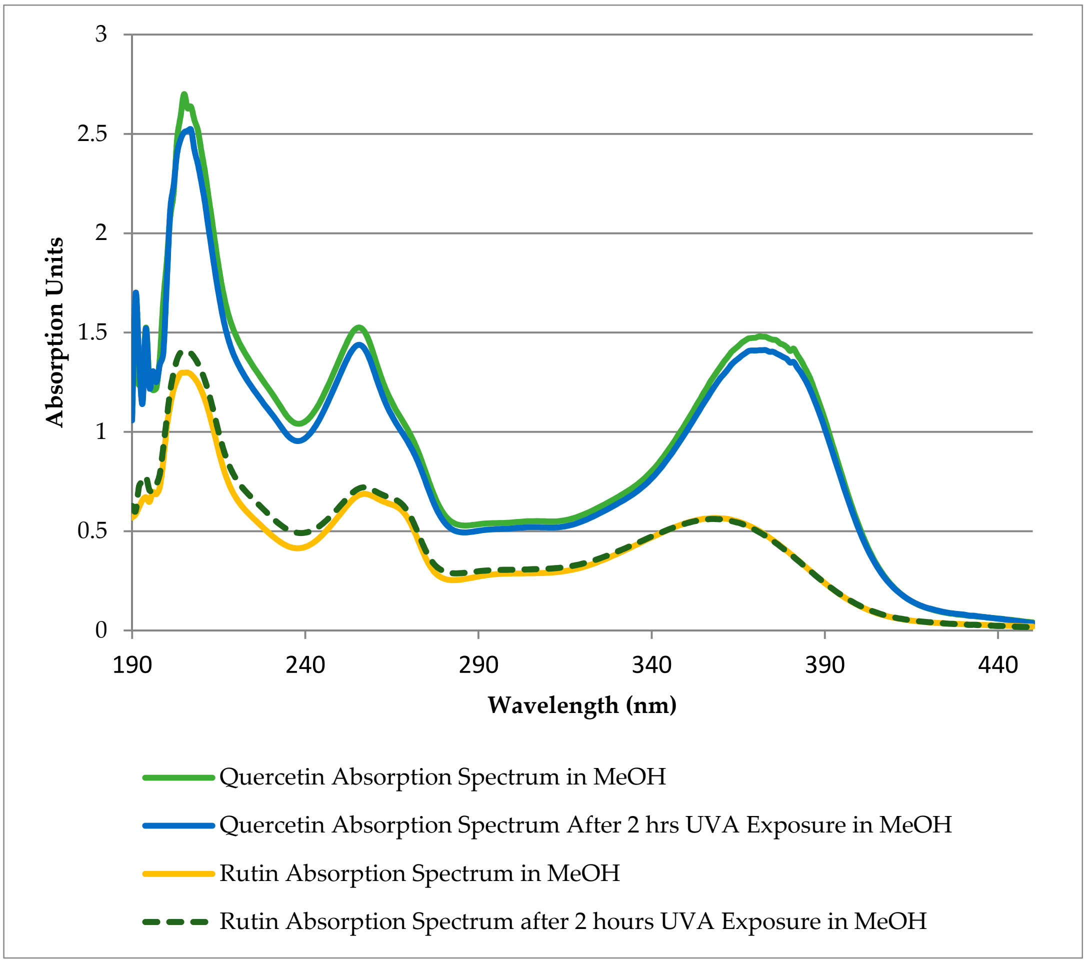

Figure S1. UV absorption spectra of quercetin (green) and rutin (red), before and after UVA exposure. Unlike nifedipine, rutin and quercetin maintain essentially the same absorption spectrum, suggesting they are more photostable. They both have a broad absorption peak similar to that of nifedipine (λ_{max} = 348 nm) supporting the idea that rutin or quercetin could compete for photon absorption with nifedipine and thereby spare nifedipine from photodegradation.

Pharmaceutics **2019**, *11*, 594; doi:10.3390/pharmaceutics11110594 www.mdpi.com/journal/pharmaceutics

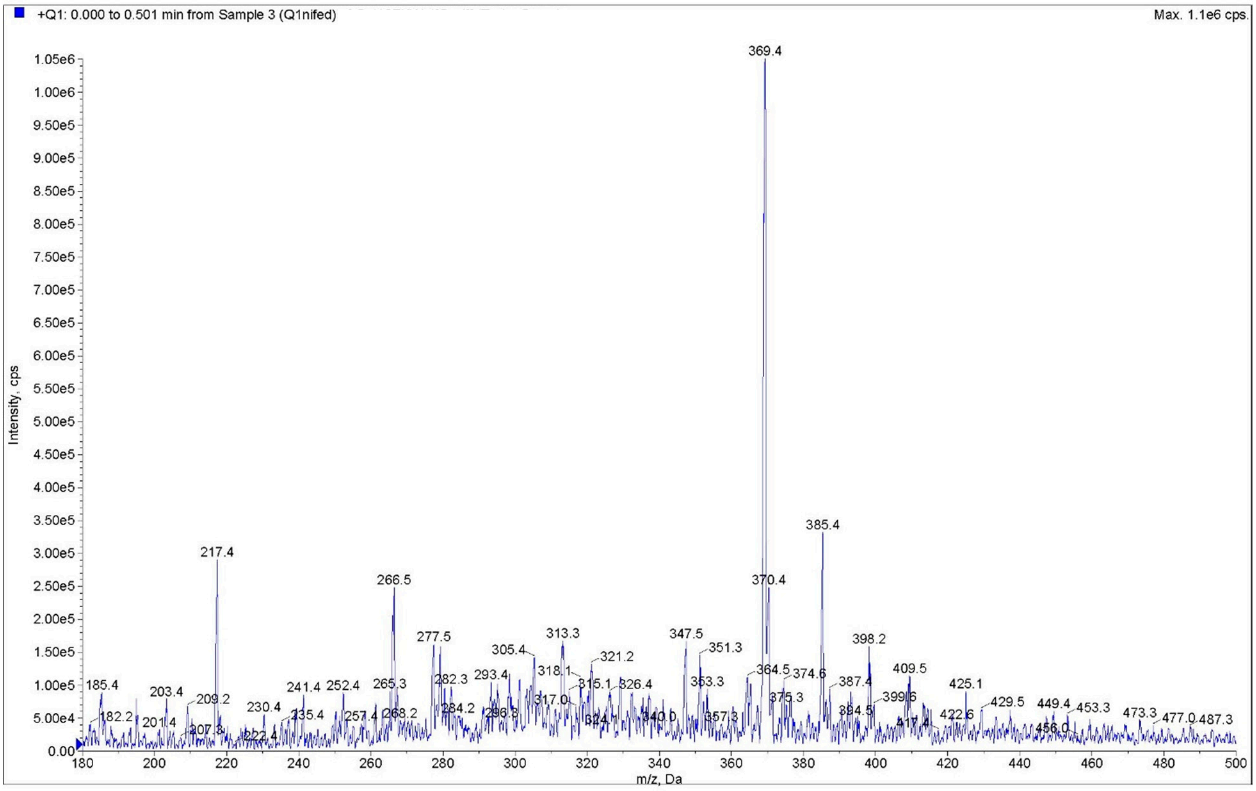

Figure S2. Mass spectrum of nifedipine extracted from the cream (Q1 scan) indicating *m*/*z* = 369.

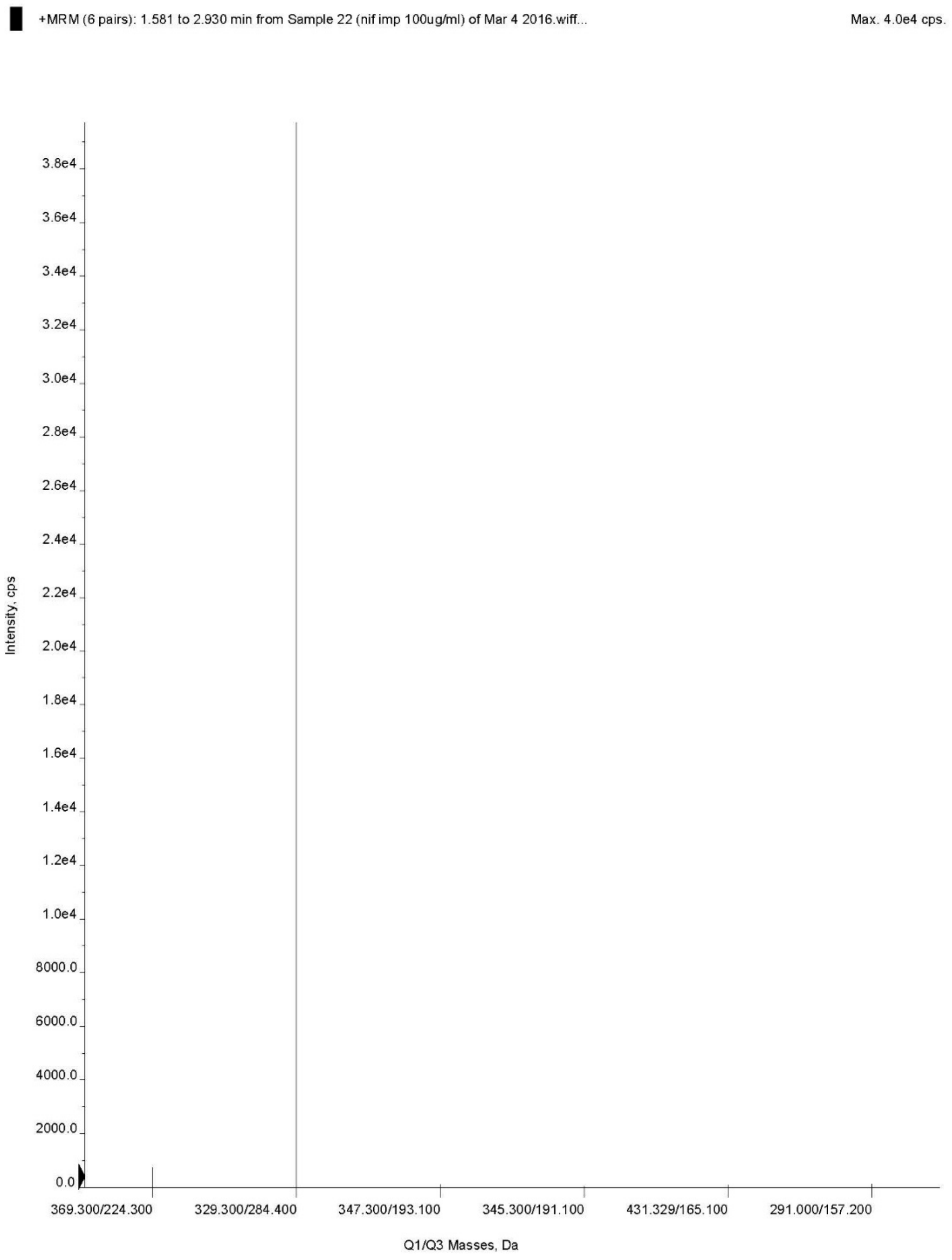

Figure S3. Mass spectrum of dehydronitrosonifedipine (+MRM) indicating *m*/*z* = 329.
